# Effect of Plasticizer Content on Mechanical and Water Vapor Permeability of Maize Starch/PVOH/Chitosan Composite Films

**DOI:** 10.3390/ma15041274

**Published:** 2022-02-09

**Authors:** Carolina Caicedo, Claudio Alonso Díaz-Cruz, Enrique Javier Jiménez-Regalado, Rocio Yaneli Aguirre-Loredo

**Affiliations:** 1 Grupo de Investigación en Química y Biotecnología (QUIBIO), Facultad de Ciencias Básicas, Universidad Santiago de Cali, Pampalinda, Santiago de Cali 760035, Colombia; carolina.caicedo03@usc.edu.co; 2 Departamento de Ingeniería Química, Facultad de Ciencias Químicas, Universidad Autónoma de Coahuila, Blvd. Venustiano Carranza SN, Saltillo 25280, Coahuila, Mexico; alonsotv12@hotmail.com; 3 Departamento de Procesos de Polimerización, Centro de Investigación en Química Aplicada (CIQA), Blvd. Enrique Reyna Hermosillo 140, Saltillo 25294, Coahuila, Mexico; enrique.jimenez@ciqa.edu.mx; 4 Investigadora por México CONACyT-CIQA, Blvd. Enrique Reyna Hermosillo 140, Saltillo 25294, Coahuila, Mexico

**Keywords:** biodegradable polymers, plasticizer, mechanical properties, water vapor permeability, starch, chitosan

## Abstract

Packaging materials based on biodegradable polymers are a viable alternative to replace conventional plastic packaging from fossil origin. The type of plasticizer used in these materials affects their functionality and performance. The effect of different plasticizers such as glycerol (GLY), sorbitol (SOR), and poly(ethylene glycol) (PEG) in concentrations of 5%, 10%, and 15% (*w*/*w*) on the structural features and functional properties of starch/PVOH/chitosan films was evaluated. The incorporation of a plasticizer increased the thickness of the biodegradable composite films. Furthermore, the material plasticized with 30% (*w*/*w*) sorbitol had the highest elongation at break, lowest water vapor permeability, and better thermal resistance. The results obtained in this study suggest that maize starch/PVOH/chitosan biodegradable composite films are a promising packaging material, and that sorbitol is the most suitable plasticizer for this formulation.

## 1. Introduction

Packaging materials are an essential part of food processing and play an important role in the food industry; thus, the development and use of new alternatives have increased. In the search for efficiency and practicality, humanity has developed packaging that, while efficient for food protection, has led to excessive use of synthetic polymers. The packaging industry consumes 36% of the synthetic plastic produced worldwide, which for 2015 was estimated at 400 million tons, of which only 9% was recycled and 79% ended up in landfills or polluting the environment [1]. Therefore, it is necessary to find a sustainable alternative that can replace synthetic plastics using biodegradable polymers. The principal natural polymers used to make biodegradable films are starch, cellulose, alginate, pectin, chitosan, zein, and gelatin [2,3,4]. Starch is a natural polymer widely used to create packaging materials because it can be obtained in high quantities and from various plant sources; however, it has much lower functional, mechanical, and barrier properties than synthetic polymers. To improve its functional performance, it is necessary to resort to the combination of two or more polymers, taking advantage of the properties or benefits of each polymer. The addition of poly(vinyl alcohol) (PVOH) in starch films improved their mechanical behavior significantly, increasing their elongation by up to 600% [5]. On the other hand, the presence of chitosan improved the hydrophobicity, water vapor barrier, and water adsorption capacity [6]. Because edible films must have characteristics such as flexibility, elasticity, and high mechanical resistance to allow their handling and maintain their physical integrity during their application, the use of plasticizing substances that improve or provide these properties is necessary. A plasticizer is a material incorporated into a polymer that helps increase flexibility and handling [7,8]. Plasticization causes an increase in the intermolecular distance and a reduction in the resistance of inter- and intramolecular forces [9]. Consequently, the movement of the polymeric chains increases, and a material with greater flexibility and increased gas permeation is obtained [9,10]. The most widely used plasticizers for biodegradable polymers are polyols, such as glycerol, propylene glycol, sorbitol, and sucrose. For adequate plasticization of packaging materials, it is necessary to consider the size, shape, and plasticizer–polymer affinity [11,12]. To obtain biodegradable packaging with mechanical performance similar to that of synthetic packaging, it is necessary to add a plasticizer that can provide the required mechanical resistance, and that does not affect its barrier capacity. The objective of this study was to determine the effects of the addition in three different concentrations of the most commonly used plasticizers in packaging materials of biodegradable origin, glycerol, sorbitol, and poly(ethylene glycol). The effect of plasticizer concentration (15%, 30%, and 45% (*w*/*w*)) on the WVP, morphology, and thermal and mechanical properties of composite films based on maize starch/poly(vinyl alcohol)/chitosan was evaluated.

## 2. Materials and Methods

### 2.1. Materials

The biopolymers used were maize starch from Meelunie B.V. (Amsterdam, The Netherlands), chitosan from Coyote Foods, Biopolymer and Biotechnology (Saltillo, Mexico), and poly(vinyl alcohol) (PVOH) (Mw 146,000–186,000, and 99% hydrolyzed) from Sigma-Aldrich (Saint Louis, MO, USA). Glycerol (J.T. Baker, Ciudad de Mexico, Mexico), poly(ethylene glycol) (average Mn 300), and d-sorbitol from Sigma (Germany and France, respectively) were used as plasticizers. Glacial acetic acid was obtained from Productos Quimicos Monterrey S.A. (Monterrey, Mexico). Sodium bromide (NaBr) and barium chloride (BaCl_2_) from Jalmek Cientifica (Monterrey, Mexico) were used to prepare supersaturated saline solutions of 54% and 90% relative humidity (RH), respectively.

### 2.2. Preparation of Filmogenic Solutions

To formulate 100 g of a filmogenic solution, 3 g of maize starch and 1 g of PVOH were dispersed in 76 mL of distilled water; then, when the solution reached 50 °C, 20 mL of chitosan (1% *w/v* in 1% *v/v* acetic acid) was added. Subsequently, the starch/PVOH/chitosan film-forming solutions were gelatinized under constant stirring at 80 °C for 80 min. At the end of that time, the amount of plasticizer was added, and it was kept under stirring for 10 min. The plasticizers, glycerol (GLY), sorbitol (SOR), and poly(ethylene glycol) (PEG), were used at concentrations of 15%, 30%, and 45% (*w*/*w*). A film without plasticizers was made as a control. The film-forming solution was dried in an acrylic mold at 65 °C for 5 h in a convection oven (Oven Series 9000, Thermolyne). The drying process and temperature were determined according to the methodology of Calambas, Heidy Lorena, Abril Fonseca, Dayana Adames, Yaneli Aguirre-Loredo and Carolina Caicedo [6], and Jiménez-Regalado, Enrique Javier, Carolina Caicedo, Abril Fonseca-García, Claudia Cecilia Rivera-Vallejo and Rocio Yaneli Aguirre-Loredo [13]. The composite films were previously conditioned for 48 h at 25 °C and 54% RH before evaluating their physicochemical properties.

### 2.3. Thickness

The thickness was determined according to the methodology used in a previous study [13] using a micrometer (Mitutoyo, model C112EXB, Aurora, IL, USA).

### 2.4. Thermogravimetric Analysis

The effect of heat treatment on the biodegradable films was determined using TGA. The thermogravimetric analyzer TGA Q500 (TA Instruments, New Castle, DE, USA) was used. The test conditions were as follows: temperature 30–600 °C at a heating rate of 10 °C·min^−1^, in a nitrogen atmosphere (50 cm^3^·min^−1^).

### 2.5. X-ray Diffraction (XRD) Analysis

To determine the order of the polymer chains of the films, a Siemens D500 powder diffractometer (Siemens Aktiengesellschaft, Munich, Germany) was used, following the methodology of Fonseca-García, Abril, Carolina Caicedo, Enrique Javier Jiménez-Regalado, Graciela Morales and Rocio Yaneli Aguirre-Loredo [14].

### 2.6. Morphology by SEM

The surface morphology of the films composed of starch/PVOH/chitosan with different plasticizers was observed using an SM-510 scanning electron microscope (TOPCON, Japan) at 15 kV. The samples were previously coated with gold–palladium for 90 s, with a working distance of 18 mm.

### 2.7. Mechanical Properties

To evaluate the mechanical behavior of the films, the tensile strength and percentage of elongation at fracture were determined, following the methodology of Jiménez-Regalado, Enrique Javier, Carolina Caicedo, Abril Fonseca-García, Claudia Cecilia Rivera-Vallejo and Rocio Yaneli Aguirre-Loredo [13]. A texture analyzer equipment (TA.XT Express, Stable Micro Systems, Godalming, UK) was used, provided with tension clamps (A/TG). The average and standard deviation of 15 samples (10 mm × 60 mm) were obtained.

### 2.8. Water Vapor Permeability (WVP)

The water vapor permeation capacity was evaluated to determine the amount of water in the gas form that passes through the films developed in this study. The water vapor permeability (WVP) test was carried out according to the ASTM E96-02 standard [15]. Circular film samples (24.64 mm diameter) were mounted on a glass cell containing silica gel. The cell was placed in a chamber with a relative humidity of 90%, with which it was possible to obtain a pressure differential of 2854.23 Pa. The weight change of the cell was monitored every 60 min for 8 h. The average and standard deviation of three replicates per formulation were obtained.

### 2.9. Statistical Analysis

An analysis of variance (ANOVA) and Tukey’s test (significance level *p* < 0.05) were performed using OriginPro 8.5.0 SR1 software (OriginLab Corporation, Northampton, MA, USA).

## 3. Results and Discussion

### 3.1. Thickness

**Figure 1:**
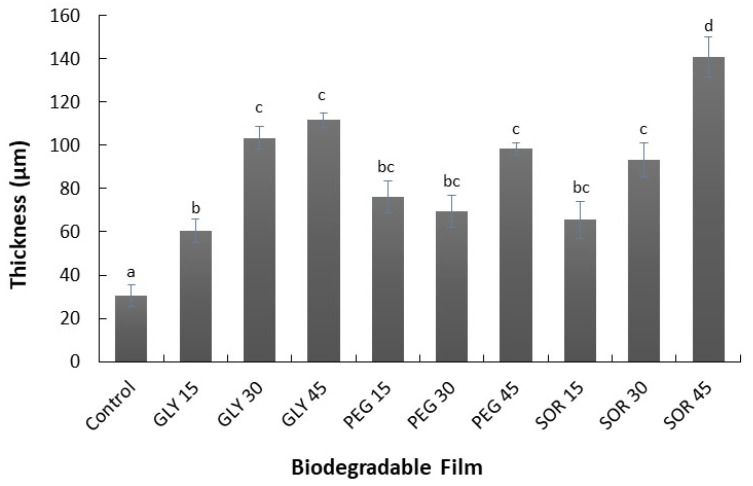
Thickness of composite films made from maize starch, PVOH, and chitosan plasticized with glycerol (GLY), sorbitol (SOR), and poly(ethylene glycol) (PEG) in ratios of 15%, 30%, and 45% (*w*/*w*). Values with a different letter denote a significant difference (Tukey test; *p* < 0.05).

Homogeneous, thin, and flexible homogeneous maize starch/PVOH/chitosan films were obtained. The amount of filmogenic solution was the same for each of the materials to keep the weight of polymers used the same. Despite maintaining the same proportion of polymers in each film, it was observed that the plasticized films changed their thickness significantly. The incorporation of the three plasticizers used, glycerol (GLY), sorbitol (SOR), and poly(ethylene glycol) (PEG), was observed to significantly increase the thickness of the biodegradable composite films (Figure 1) when compared with the control film without plasticizing, which had a thickness of 31 ± 5 µm. The thicknesses of the glycerol-plasticized composite films ranged from 60 ± 6 µm (GLY15) to 111 ± 3 µm (GLY45), while those of the poly(ethylene glycol)-plasticized films ranged from 76 ± 7 µm (PEG15) to 98 ± 3 µm (PEG45). Sorbitol was the plasticizer that increased the thickness of the films the most, from 65 ± 8 to 141 ± 9 µm when the plasticizer content increased from 15% to 45% (*w*/*w*). This change in thickness caused by the increase in the content of these plasticizers was similar to that reported in other starch-based films such as sugar palm [16] and anchote tuber [17].

### 3.2. Thermogravimetric Analysis (TGA)

**Figure 2:**
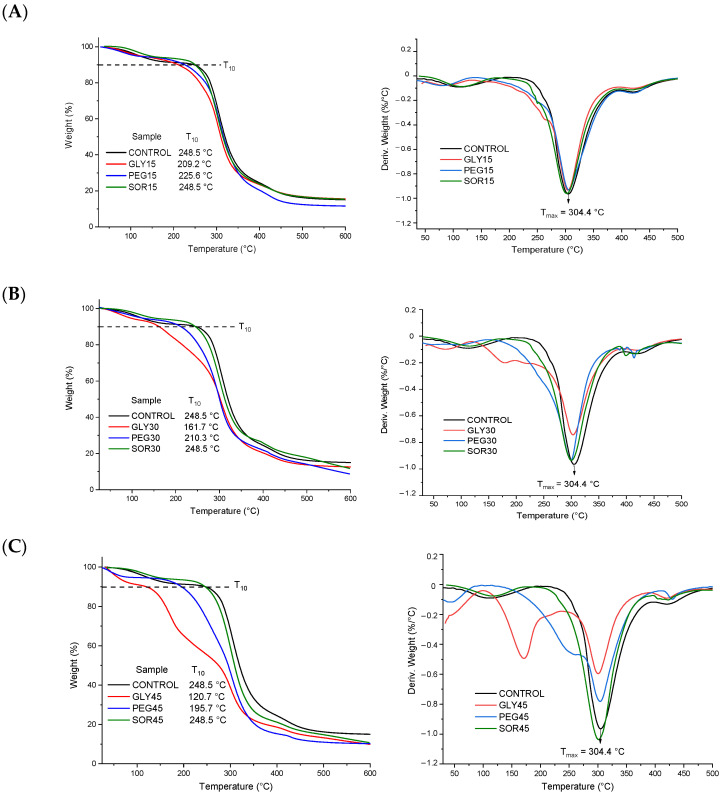
TGA curves of biopolymeric blends plasticized with (**A**) 15%, (**B**) 30%, and (**C**) 45% (*w*/*w*). Plasticizers used were glycerol (GLY), polyethylene glycol (PEG), and sorbitol (SOR).

In the thermal analysis curves (Figure 2), it was observed that the three polymers exhibited degradation around 300 °C. The degradation temperature (Tmax) for the control film (starch/PVOH/chitosan) was consistent with data previously reported in the literature for each polymer [5,13]. In general, it was observed that, when incorporating 15% (*w*/*w*) of the different plasticizers, two thermal events resulted: the first, around 120 °C, represented the loss of humidity (see Figure 2A); the second, at approximately 305 °C, represented the degradation of the plasticized polymer blend. Figure 2B shows the behavior of the biopolymeric blends that incorporated 30% (*w*/*w*) of the different plasticizers; in this case, GLY30 exhibited a marked instability at ~175 °C, with complete loss of the plasticizer before 300 °C (when the degradation of polymers occurred). A similar effect was observed in PEG30. The sample SOR30 conserved the thermal behavior of SOR 15%. Lastly, the samples with 45% (*w*/*w*) (Figure 2C) addition of the plasticizer presented the following order of thermal stability: control ≈ SOR45 > PEG45 > GLY45.

In general, it was shown that GLY and PEG in concentrations ≥30% resulted in a decrease in the plasticization of the biopolymer blend by inducing an anticipated thermal event (175 °C). In contrast, SOR 15%, 30%, and 45% (*w*/*w*) presented TGA curves practically superimposed and almost equal to the control, indicating its better interaction with all polymers. The behavior of the blends was largely related to the type and concentration of plasticizer used, as presented in previous investigations with GLY and SOR [18]. Interaction models have been proposed through hydrogen bridges between hydroxyl and amino groups of the components [19]. It has been explained that glycerol, due to its smaller size, achieves faster absorption and desorption processes. On the other hand, sorbitol promotes stronger interactions with polar molecules (hydrophilic) due to the presence of more –OH groups. In this way, the plasticization of the starch/PVOH/chitosan blends is favored by a greater participation of hydroxyl in the structure. Additionally, at concentrations equal to 15% (*w*/*w*) for GLY, PEG, and SOR, the latter also favored thermal stability (up to 45% (*w*/*w*)).

### 3.3. X-ray Diffraction (XRD)

**Figure 3:**
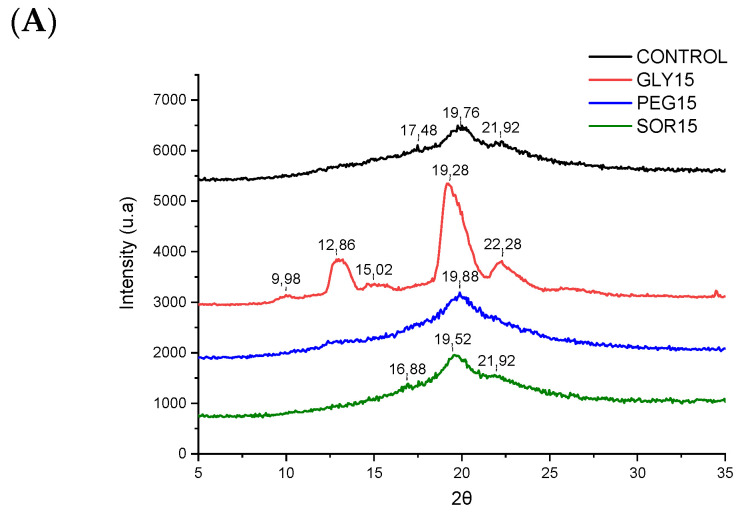

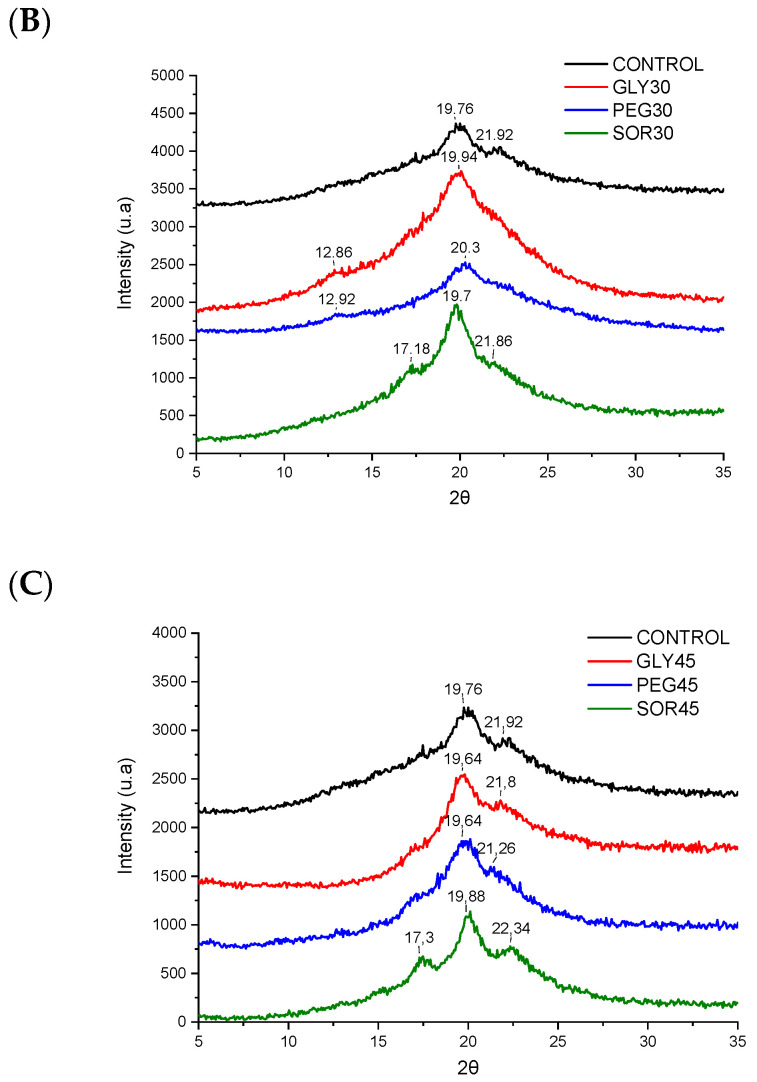
XRD patterns of biopolymeric blends plasticized with (**A**) 15%, (**B**) 30%, and (**C**) 45% (*w*/*w*).

Figure 3 shows the XRD pattern, which was used to determine the crystalline structure of the plasticized biopolymeric blends. Unplasticized maize starch with amylose percentages <40% showed characteristic peaks with respect to the type A crystalline structure in its XRD pattern, at angles that could be observed at 14.9°, 16.9°, 17.8°, 19.7°, 22.9°, and 30.3° [20]. Gelatinized starch presented an XRD pattern where the peaks at angles of 13.5° and 20.8° predominated [21]. This is because type A crystals (native crystallinity typical of cereals) are replaced by type V crystals (induced crystallinity during processing) [22], which are formed by the rapid crystallization of amylose in simple helices. This process involves a strong interaction with polar molecules during shear/heating and subsequent cooling [23]. In the case of PVOH crystals, one of the characteristic peaks was observed at an angle of 19.8°, which shifted to a lower angle (~19.7°) upon interaction with starch. This particular peak corresponds to the crystallographic plane (101) which represents a semicrystalline structure due to the interaction via hydrogen bonds between the hydroxyl groups (–OH) [24]. On the other hand, chitosan usually presents two characteristic peaks 2θ, at 9.4° and 20.0°, of crystalline forms I and II, respectively [25]. Figure 3A shows a comparison of the different plasticizers incorporated at 15% (*w*/*w*). GLY15 exhibited a different structural ordering from the control sample, PEG15, and SOR15. GLY15 presented peaks corresponding to the crystallographic patterns of the components independently, as a polyphasic sample typical of a nonhomogeneous mixture. Unlike the GLY30 and GLY45 samples, a decrease in intensity was observed in the peaks, while there was widening around the 19.7° peak and a shift at higher angles (from 19.2° to 19.9°). This can be explained by the formation of new crystalline phases containing the chains between starch/PVOH/chitosan. Some authors reported similar results from biopolymeric mixtures where they showed co-crystal formation [26]. Regarding the incorporation of 30% and 45% (*w*/*w*) (Figure 3B,C) plasticizers in the biopolymeric blend, the formation of a greater presence of crystalline domains was observed in the samples with plasticizers of a smaller atomic order.

### 3.4. Morphology by SEM 

**Figure 4:**
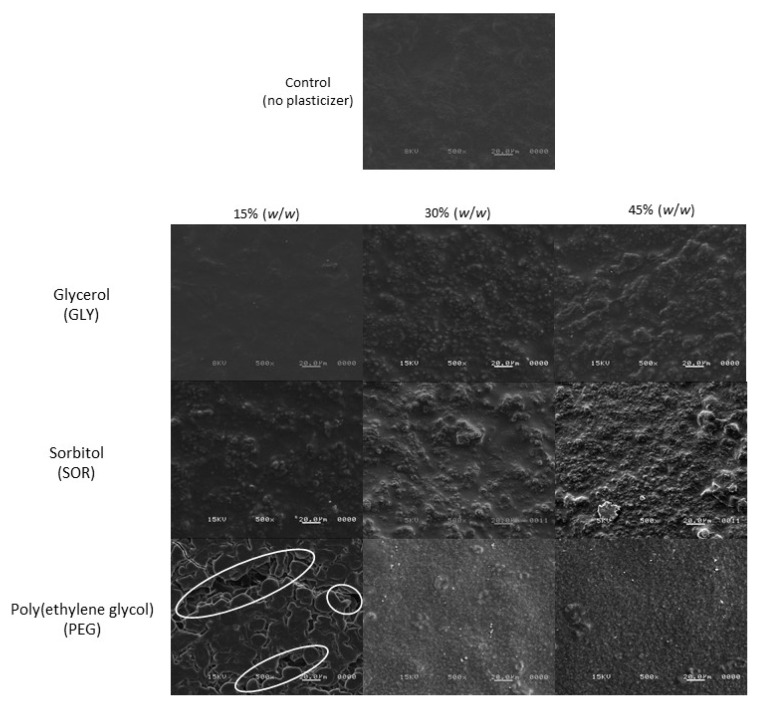
Surface morphology of biodegradable starch/PVOH/chitosan films plasticized with glycerol (GLY), sorbitol (SOR), and poly(ethylene glycol) (PEG) in ratios of 0% (control), 15%, 30%, and 45% (*w*/*w*). Micrographs are at a magnification of 500×.

The morphology of starch/PVOH/chitosan films is shown in Figure 4. The surface of the composite films was very different for each of the plasticizers used. Likewise, a change was also observed in the surface morphology of films with the same plasticizer at different concentrations. The films that presented a less rough surface, with a similar appearance to the control sample, as well as fewer changes with the increase in plasticizer, were those that were plasticized with glycerol. The materials plasticized with sorbitol, as well as with PEG, presented a more irregular surface. Various agglomerations of material (probably polymer) were observed caused by poor structuring of the material (marked with a white oval in Figure 4), especially when the film was plasticized with PEG at low concentrations (15% *w*/*w*), an effect that was avoided by increasing the content of this plasticizer to 45% (*w*/*w*). The morphology of the films developed in this study was similar to that reported for purple yam starch/chitosan films and glycerol [27].

### 3.5. Mechanical Behavior

**Figure 5:**
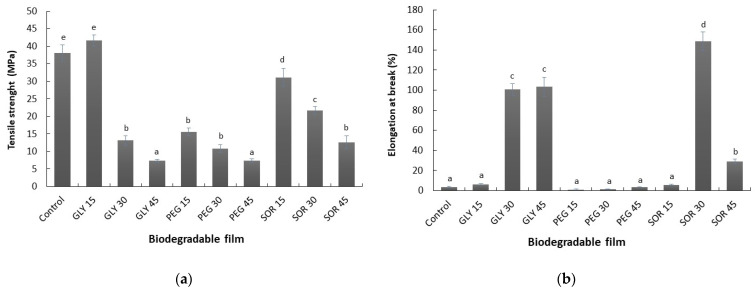
Tensile strength (**a**) and percentage of elongation at break (**b**) of biodegradable starch/PVOH/chitosan films plasticized with glycerol (GLY), sorbitol (SOR), and poly(ethylene glycol) (PEG) in concentrations of 15%, 30%, and 45% (*w*/*w*). Values with a different letter denote a significant difference (Tukey test; *p* < 0.05).

The tensile strength (TS) and elongation at break (%E) properties of starch/PVOH/chitosan composite films with the plasticizers used at the different concentrations (15%, 30%, and 45% *w*/*w*) are presented in Figure 5. It can be clearly observed that the tensile strength of the composite films decreased as the plasticizer concentration increased from 15% to 45% (*w*/*w*) (Figure 3A), regardless of the plasticizer employed, when compared to the control film. A slight relationship was observed between the thickness of the materials and the mechanical resistance. A thicker film resulted in a lower tensile strength, especially when the plasticizer concentration was 30% (*w*/*w*) or more.

The control film was found to have the highest tensile strength (38.1 ± 2.2 MPa) (Figure 3A) and a low %E (3.7% ± 0.4%) (Figure 5b), a typical pattern for brittle materials [17]. A similar result was also observed for the glycerol plasticized film at a concentration of 15% (*w*/*w*). Among the films, the PEG-plasticized films were less strong and less flexible at all plasticizer contents. In contrast, the film plasticized with 30% (*w*/*w*) sorbitol had the highest %E (148.5% ± 9.2%) (Figure 5b) and an intermediate tensile strength (21.7 ± 1.1 MPa). The presence of a plasticizing agent increases the free volume or molecular mobility within the structural matrix of the polymer by reducing the proportion of hydrogen bonding between the chains, replacing the polymer–polymer interactions with polymer–plasticizer–polymer bonds [28]. This reduction in the number of direct interactions also causes a reduction in the proximity between the polymer chains. Therefore, under tension forces, the movements of the chains are facilitated, lowering the glass transition temperature of these materials and improving their flexibility [29,30]. The gel theory could help to understand the effect observed in this study. The polymer molecules in the film-forming solution try to stay bonded to each other. Water and plasticizer molecules compete for the binding sites of the polymers, reducing the number available for the polymers to bind to. Therefore, the rigidity of the structural matrix is reduced, which is reflected in a decrease in mechanical and thermomechanical performance of the resulting materials [31].

### 3.6. Gas Permeability

**Figure 6:**
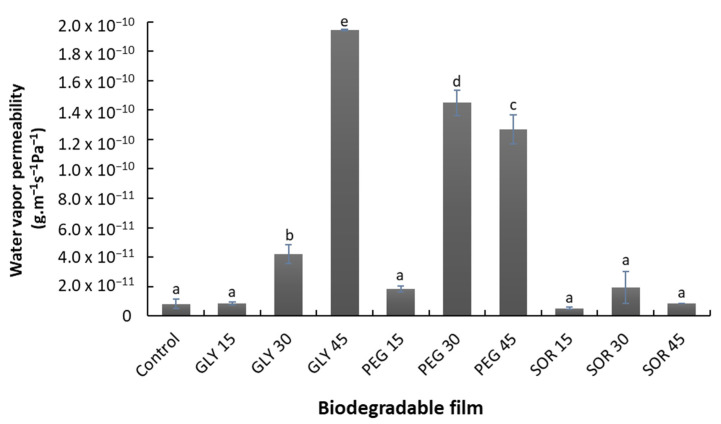
Water vapor permeability (WVP) of biodegradable composite starch/PVOH/chitosan films plasticized with glycerol (GLY), sorbitol (SOR), and poly(ethylene glycol) (PEG) in ratios of 0% (control), 15%, 30%, and 45% (*w*/*w*). Values with a different letter denote a significant difference (Tukey test; *p* < 0.05).

The water vapor permeability (WVP) of the composite films based on starch/PVOH/chitosan is presented in Figure 6; the control film presented a value of 8.11 × 10^−12^ g·m^−1^·s^−1^·Pa^−1^. The amount of gas that penetrates the material increased with increasing concentrations of plasticizers (glycerol and PEG), with glycerol increasing the most, with values from 8.3 ± 0.9 to 194.4 ± 0.5 × 10^−12^ g·m^−1^·s^−1^·Pa^−1^ when the concentration increased from 15% to 45% (*w*/*w*). 

The use of sorbitol to plasticize the films made in this study did not significantly modify the rate of gas that permeates through the material, being the best alternative for plasticizing this material. The WVP of films plasticized with sorbitol ranged from 5.2 ± 0.6 × 10^−12^ g·m^−1^·s^−1^·Pa^−1^ (SOR15) to 8.6 ± 0.2 × 10^−12^ g·m^−1^·s^−1^·Pa^−1^ (SOR45), being the films plasticized with sorbitol those that presented the lowest values. A similar behavior was observed in biodegradable films from mucilage [32], babassu starch [33], whey protein [34], and *Artemisia* gum [35]. The increase in free volume in the polymeric network and the decrease in the direct interactions between the chains also modify the gas diffusion behavior through the matrix [11,28]. Consequently, the polymer networks become less dense, promoting the adsorption of water molecules on the surface of the film (higher solubility) and easier penetration through its structure (higher diffusivity), resulting in increased WVP [30,36]. This increase in chain movement was also reflected in the results of the mechanical properties (Figure 5). The ability to reduce water vapor permeation presented by the materials obtained in this study was better than that reported for starch, pectin, and chitosan–zein films [16,37,38].

## 4. Conclusions

This study made it possible to obtain biodegradable films via the casting technique using a formulation with three biopolymers, corn starch, polyvinyl alcohol, and chitosan. The plasticizing capacity of three different plasticizing agents (glycerol, sorbitol, and PEG) was evaluated. All the plasticizers evaluated caused an improvement in the mechanical performance and gaseous water barrier of the composite films. The structural analysis of the starch/PVOH/chitosan blend revealed a decrease in the crystallinity of the composite films, with no well-defined peaks in the diffractogram attributable to the crystal structures of the independent biopolymers. The presence of the different types and concentrations of plasticizers promoted appreciable structural changes in the composite. The strong interactions through hydrogen bonds with sorbitol not only limited the movements of the molecular chain segments, but also caused the containment of the crystallization process. Additionally, the strong intermolecular interactions involving the different plasticizers up to 15%, as well as sorbitol up to 45% (*w*/*w*), of the starch/PVOH/chitosan-based biopolymer blend increased the thermal stability of the composite. The film plasticized with 30% (*w*/*w*) sorbitol presented the best mechanical performance, as well as a better barrier to water vapor and improved thermal resistance, making this material an excellent option to be used as a sustainable packaging alternative. 

## Data Availability

The data are available on request from the corresponding author.
